# Physiopathology of SARS-CoV-2-infection-associated thrombosis

**DOI:** 10.1590/1677-5449.200128

**Published:** 2020-09-21

**Authors:** Ivan Benaduce Casella

**Affiliations:** 1 Universidade de São Paulo – USP, Faculdade de Medicina, Hospital das Clínicas, São Paulo, SP, Brasil.

The pandemic provoked by the novel coronavirus (SARS-CoV-2) is the most severe public health event of recent decades, with more than 11.1 million cases registered worldwide up to the start of July 2020 and more than 528 thousand deaths.^1^ There are many different clinical manifestations of infection by SARS-CoV-2 and the pathophysiologic processes that provoke them are also numerous and varied. Since the early stages of the pandemic, the number of thrombotic events of varying types has attracted the attention of physicians and researchers, invoking the need to understand them in order to avert the most somber of clinical outcomes.

The modest ambition of this editorial is to present the scientific theories and evidence on the pathophysiology of thrombotic processes associated with infection by SARS-CoV-2. However, certain important provisos should be made clear: part of these scientific explanations have not yet progressed beyond the status of hypotheses; the pathological processes observed rather than sequential, are predominantly concomitant and stimulate each other; it is difficult to quantify the impact of events that take place on the molecular and cellular scales have on the final clinical outcomes of the thrombotic event.

## THE ROLE OF ANGIOTENSIN-CONVERTING ENZYME TYPE 2 (ACE2)

The principal mechanism that SARS-CoV-2 has for accessing the intracellular environment is through interaction between its S surface glycoprotein and the human ACE2 glycoprotein, which is present both in plasma and in the membranes of several different cell types.^2^ ACE2 acts as a membrane receptor in this process, which also involves interactions with other effectors, such as type 2 transmembrane serine proteases.^3^

The ACE2 protein plays an important role as a negative effector in the renin angiotensin aldosterone system, converting angiotensins I and II into angiotensins 1-9 and 1-7, respectively. Angiotensins 1-9 and 1-7 have vasodilatory and anti-inflammatory effects, among other actions, thereby antagonizing the classic hypertensive and inflammatory effects of angiotensin II.^2^^,^^3^

Infection by SARS-CoV-2 causes death of cells rich in ACE2 receptors and cellular internalization of a proportion of these receptors, ultimately causing a reduction in circulating ACE2 activity.^4^ This results in angiotensin II activity predominating over activity of angiotensins 1-7 and 1-9. In addition to its hypertensive and inflammatory effects, angiotensin II stimulates activation of the coagulation cascade via the tissue factor (TF) pathway, elevates activity of tissue plasminogen activator inhibitor type 1 (PAI-1) and inhibits expression of tissue plasminogen activator (tPA).^5^

## IMMUNOTHROMBOSIS AND NEUTROPHIL EXTRACELLULAR TRAPS

The term “immunothrombosis” has been used to describe the interactions between macrophages, polymorphonuclear cells, platelets, coagulation factors, and immunoeffector proteins, forming thrombi in the microvasculature which function to recognize pathogens and mechanically preventing them from propagating.^6^ Immunothrombosis is usually triggered by infectious agents circulating in the blood and can be activated by viral infections.

The polymorphonuclear cells involved in this process stimulate formation of neutrophil extracellular traps, which can stimulate coagulation activation via factor XII and also act to inhibit endogenous anticoagulant proteins.^5^ Zuo et al. observed that elevated levels of neutrophil activation and formation of neutrophil extracellular traps in patients positive for Covid (Covid+) were associated with an increased risk of thrombotic complications.^7^

## ENDOTHELIAL RESPONSE, INFLAMMATION, AND THROMBOSIS

Ackermann et al.^8^ demonstrated that pulmonary infection by SARS-CoV-2 is associated with microthrombosis, endotheliitis, and intussusceptive angiogenesis, at an intensity not observed in other viral infections or interstitial pneumonia of similar severity. Under physiological conditions, the vascular endothelium has a variety of different mechanisms for inhibiting thrombotic events. Conversely, pathological stimuli at the cellular or molecular level stimulate thrombogenic endothelial responses, such as increased expression of FT and of PAI-1, platelet activation, release of inflammatory cytokines and reduced expression of endogenous anticoagulants, such as thrombomodulin.^9^

These inflammatory cytokines are also massively secreted by alveolar macrophages and epithelial and polymorphonuclear cells^10^ provoked by the exaggerated interferon-mediated late immune response.^11^ The vicious circle of inflammation and thrombosis is reignited by cytokines, which are chemotactic factors for leukocytes, activate coagulation via tissue factor, inhibit fibrinolysis by increasing expression of PAI-1, and inhibit endogenous anticoagulant pathways, such as antithrombin, protein C, and its cofactor, protein S.^10^^,^^12^

The complement system is an element in the immune response to SARS-CoV-2 infection and is also associated with the thrombotic processes observed. Magro et al.^13^ observed intense complement system activity in critical patients who were positive for SARS-CoV-2, with deposition of C5b-9 and C4d fractions in the microvasculature, associated with microthrombosis with deposition of fibrin and endothelial injury.

## D DIMER

Marked D dimer elevation has been observed in Covid+ patients, with extremely high levels in the most severe patients and clear associations with poor prognosis.^14^ While the phenomenon is not entirely understood, it may be related to occurrence of massive microvascular thrombosis and activation of the fibrinolytic system by direct viral stimulation, followed by thrombolysis inhibition.^14^^,^^15^

In summary, we can group the effects of the events described into four main results that act on coagulation: (1) activation of the coagulation cascade by different routes and multiple stimuli of different origins; (2) platelet activation; (3) inhibition of endogenous anticoagulant proteins (protein C and its cofactor protein S, antithrombin, and tissue factor pathway inhibitor); and (4) inhibition of the fibrinolytic system (fibrinolysis shutdown).

## SARS-CoV-2 INFECTION, THROMBOTIC EVENTS, AND HEPARIN RESISTANCE

Several authors^16^^-^18 have reported increased rates of thrombotic events, in both the venous (deep venous thrombosis and pulmonary embolism) and arterial systems (ischemic strokes and peripheral arterial thromboses) of patients infected by SARS-CoV-2. The methods used in these reports differ considerably, preventing compilation of homogenous data. Helms et al.^19^ observed increased incidence of venous thromboembolism in critical patients infected by SARS-CoV-2 in comparison with patients with severe respiratory infections caused by other pathogens.

White et al.^20^ observed a small cohort of patients infected by SARS-CoV-2 who were given therapeutic anticoagulation with heparins. In this group, 5 out of 5 patients treated with enoxaparin exhibited lower anti-factor Xa activity than required for the drug to have therapeutic effects. With unfractionated heparin, 8 out of 10 patients exhibited resistance to anticoagulation.

In a similar manner, Dutt et al.^21^ noted that 27% of patients with non-severe SARS-CoV-2 infection who were given prophylactic doses of heparin had anti-factor Xa activity below the level indicative of prophylaxis efficacy. Among the more serious patients, in intensive care, the percentage was 95%.

## CONCLUSIONS

Current evidence indicates that there is an elevated incidence of clinically relevant thrombotic events is associated with SARS-CoV-2 infection. Understanding of the pathophysiologic processes that cause these thrombotic phenomena is still incomplete. The cause-effect relationship between thrombotic events and the severity of SARS-CoV-2 infection also needs to better understood. It is not clear whether it is patients with greater thrombotic response who have more severe clinical presentations, or whether the process follows the opposite sequence, by which cases of greater clinical severity have a higher incidence of thrombotic events.
