# Supplementary material for: Physiopathology of SARS-CoV-2-infection-associated thrombosis
Source: J Vasc Bras. 2020 Sep 21;19:e20200128. doi: 10.1590/1677-5449.200128 (PMC8218000; doi:10.1590/1677-5449.200128)
Supplement: Translation

# Translation: Fisiopatologia da trombose associada à infecção pelo SARS-CoV-2

**Authors:** Ivan Benaduce Casella

**Affiliations:** 1 Universidade de São Paulo – USP, Faculdade de Medicina, Hospital das Clínicas, São Paulo, SP, Brasil.

A pandemia provocada pelo novo coronavírus (SARS-CoV-2) é o evento mais severo de saúde pública das últimas décadas, com mais de 11,1 milhões de casos contabilizados em todo o mundo até o início de julho de 2020 e mais de 528 mil mortes^1^. As manifestações clínicas da infecção pelo SARS-CoV-2 são múltiplas, assim como os processos fisiopatológicos que as proporcionam. Desde o início, o número de eventos trombóticos de diversas naturezas tem chamado a atenção de médicos e pesquisadores, evocando a necessidade de sua compreensão para que os desfechos clínicos mais sombrios sejam evitados.

Este editorial tem a modesta pretensão de apresentar as teorias e evidências científicas acerca da fisiopatologia dos processos trombóticos associados à infecção pelo SARS-CoV-2. No entanto, há alguns aspectos importantes a serem realçados: parte das explicações cientificas ainda se encontram no campo das hipóteses; os processos patológicos observados são predominantemente concomitantes e se estimulam mutuamente; ainda é difícil quantificar o impacto isolado de eventos que ocorrem em escala molecular e celular nos desfechos clínicos finais do evento trombótico.

## PAPEL DA ENZIMA DE CONVERSÃO DA ANGIOTENSINA TIPO 2 (ECA2)

O principal mecanismo de acesso do SARS-CoV-2 ao meio intracelular se dá pela interação de sua glicoproteína de superfície S com a glicoproteína humana ECA2, presente tanto no plasma como na membrana de diversos tipos celulares^2^. Nesse processo, a ECA2 atua como receptor de membrana, contando ainda com a interação de outros efetores, como a serina protease transmembrana tipo 2^3^.

A proteína ECA2 cumpre importante papel de efetor negativo no sistema renina angiotensina aldosterona, convertendo as angiotensinas I e II em angiotensinas 1-9 e 1-7, respectivamente. As angiotensinas 1-9 e 1-7 apresentam efeitos vasodilatadores e anti-inflamatórios, dentre outros, antagonizando, assim, os efeitos clássicos hipertensivos e inflamatórios da angiotensina II^2^^,^^3^.

A infecção pelo SARS-CoV-2 resulta em morte de células ricas em receptores ECA2 e na internalização celular de parte desses receptores, causando, em última instância, redução da atividade da ECA2 circulante^4^. Com isso, há um predomínio da atividade da angiotensina II sobre as angiotensinas 1-7 e 1-9. A angiotensina II, além de seu efeito hipertensor e inflamatório, estimula a ativação da cascata da coagulação pela via do fator tissular (FT), eleva a atividade do inibidor do ativador do plasminogênio tecidual tipo 1 (PAI-1) e inibe a expressão do ativador do plasminogênio tecidual (tPA)^5^.

## IMUNOTROMBOSE E REDES EXTRACELULARES DE NEUTRÓFILOS

O termo “imunotrombose” tem sido utilizado para descrever a interação entre macrófagos, polimorfonucleares, plaquetas, fatores de coagulação e proteínas imunoefetoras, formando trombos na microvasculatura com o intuito de identificar patógenos e restringir mecanicamente a sua propagação^6^. A imunotrombose é usualmente desencadeada por agentes infecciosos circulantes no sangue, podendo também ser acionada em infecções virais.

Os polimorfonucleares envolvidos nesse processo estimulam a formação de redes extracelulares de neutrófilos, que podem estimular a ativação da coagulação via fator XII atuando também na inibição de proteínas anticoagulantes endógenas^5^. Zuo et al. observaram que elevados níveis de ativação de neutrófilos e formação de redes extracelulares de neutrófilos em pacientes positivos para Covid (Covid+) foram associados a maior risco de complicações trombóticas^7^.

## RESPOSTA ENDOTELIAL, INFLAMAÇÃO E TROMBOSE

Ackermann et al.^8^ demonstraram que infecção pulmonar pelo SARS-CoV-2 está associada a microtrombose, endotelite e angiogênese intussusceptiva, em uma intensidade não observada em outras infecções virais ou pneumonias intersticiais de semelhante gravidade. O endotélio vascular em condições fisiológicas dispõe de diversos mecanismos inibidores de eventos trombóticos. De forma oposta, estímulos patológicos em nível celular ou molecular estimulam uma resposta trombogênica endotelial, como o aumento da expressão do FT e do PAI-1, ativação plaquetária, liberação de citocinas inflamatórias e redução de expressão de anticoagulantes endógenos, como a trombomodulina^9^.

Essas citocinas inflamatórias também são maciçamente liberadas por macrófagos alveolares, células epiteliais e polimorfonucleares^10^ pela resposta imunológica tardia e exacerbada mediada por interferons^11^. Realimentando o círculo vicioso da inflamação e trombose, as citocinas são fatores quimiotáticos para leucócitos, ativam a coagulação pela via do fator tissular, inibem a fibrinólise ao elevar a expressão do PAI-1 e inibem vias anticoagulantes endógenas, como a antitrombina, a proteína C e seu cofator, a proteína S^10^^,^^12^.

O sistema complemento é um dos elementos da resposta imune na infecção pelo SARS-CoV-2 e também está associado aos processos trombóticos observados. Magro et al.^13^ observaram intensa atividade do sistema complemento em pacientes críticos positivos para SARS-CoV-2, com deposição de frações C5b-9 e C4d na microvasculatura, associadas à microtrombose com deposição de fibrina e lesão endotelial.

## DÍMERO D

A marcante elevação do dímero D foi observada em pacientes Covid+, sendo superlativa nos pacientes mais graves e claramente associada a piores prognósticos^14^. Embora não totalmente compreendida, pode ser explicada pela ocorrência de maciça trombose microvascular e pela ativação do sistema fibrinolítico por estímulo viral direto com posterior inibição deste^14^^,^^15^.

Resumidamente, podemos agrupar os efeitos dos eventos descritos em quatro resultantes principais agindo sobre a coagulação: (1) ativação da cascata da coagulação por vias distintas e múltiplos estímulos de origens distintas; (2) ativação plaquetária; (3) inibição das proteínas anticoagulantes endógenas (proteína C e seu cofator proteína S, antitrombina, inibidor da via do fator tissular); (4) inibição do sistema fibrinolítico (“*fibrinolysis shutdown*”).

## INFECÇÃO PELO SARS-COV-2, EVENTOS TROMBÓTICOS E RESISTÊNCIA À HEPARINA

Diversos autores^16^^-^18 reportaram aumento de eventos trombóticos tanto venosos (trombose venosa profunda, embolia pulmonar) quanto arteriais (acidente vascular encefálico isquêmico, tromboses arteriais periféricas) em pacientes infectados pelo SARS-CoV-2. Tais relatos diferem bastante em seus métodos, o que impede a obtenção de dados homogêneos. Helms et al.^19^ observaram incidência aumentada de eventos de tromboembolismo venoso em pacientes graves infectados pelo SARS-CoV-2 em comparação com pacientes com infecções respiratórias severas causadas por outros patógenos.

White et al.^20^ observaram um pequeno coorte de pacientes infectados pelo SARS-CoV-2 recebendo anticoagulação terapêutica com heparinas. Neste grupo, 5 em 5 pacientes tratados com enoxaparina apresentaram níveis de atividade antifator Xa inferiores aos esperados para ação terapêutica da droga. Para a heparina não fracionada, 8 em 10 pacientes apresentaram resistência à anticoagulação.

De maneira similar, Dutt et al.^21^ notaram que 27% dos pacientes com infecção não severa pelo SARS-CoV-2 recebendo doses profiláticas de heparina apresentavam valores de atividade antifator Xa abaixo dos níveis indicadores de efetividade da profilaxia. Já nos pacientes mais graves, internados em ambiente de terapia intensiva, a porcentagem era de 95%.

## CONCLUSÕES

As evidências atuais indicam que existe uma expressiva incidência de eventos trombóticos clinicamente relevantes associados à infecção pelo SARS-CoV-2. A compreensão dos processos fisiopatológicos que resultam em tais fenômenos trombóticos ainda é incompleta. Também, a relação de causa e efeito entre eventos trombóticos e a gravidade da infecção pelo SARS-CoV-2 ainda precisa ser mais bem compreendida. Não está claro se são os pacientes com maior resposta trombótica que apresentam os quadros clínicos mais severos ou se o processo é sequencialmente oposto, em que os casos clinicamente mais graves resultam em maior incidência de eventos trombóticos.
